# Molecular classification of endometrial cancer in fertility-sparing-treatment: toward a personalized clinical approach

**DOI:** 10.3389/fonc.2026.1886770

**Published:** 2026-09-03

**Authors:** Filippo Alberto Ferrari, Giorgio Bogani, Matteo Pavone, Elena Olearo, Andrea Puppo, Marcello Ceccaroni

**Affiliations:** 1 Department of Obstetrics and Gynecology, Gynecologic Oncology and Minimally Invasive Pelvic Surgery, International School of Surgical Anatomy (ISSA), IRCCS “Sacro Cuore - Don Calabria” Hospital, Verona, Italy; 2 Gynecological Oncology Unit, Fondazione IRCCS Istituto Nazionale dei Tumori di Milano, Milan, Italy; 3 IHU Strasbourg, Institute of Image-Guided Surgery, Strasbourg, France; 4 Dipartimento di Scienze per la Salute della Donna e del Bambino e di Sanità Pubblica, IRCCS, UOC Ginecologia Oncologica, Fondazione Policlinico Universitario A. Gemelli, Rome, Italy; 5 IRCAD, Research Institute Against Digestive Cancer, Strasbourg, France; 6 Gynecology and Obstetrics Unit, Santa Croce e Carle Hospital, Cuneo, Italy

**Keywords:** endometrial cancer, fertility sparing, hormonal therapy, molecular classification, progestin

## Abstract

Molecular classification has emerged as a promising tool to improve patient selection and prognostic stratification in fertility sparing treatment (FST) for endometrial cancer (EC). Although FST is currently considered a valid option for carefully selected patients with early stage, low grade endometrioid EC, recurrence rates remain substantial and conventional clinicopathologic criteria inadequately capture tumor biological heterogeneity. Current evidence suggests that NSMP tumors represent the most favorable subgroup for FST, showing the highest complete response rates and lowest recurrence rates, likely due to preserved estrogen and progesterone receptor signaling. In contrast, MMRd/MSI H and p53abn tumors demonstrate poorer oncologic outcomes, reduced sensitivity to progestin therapy, and higher recurrence rates. Although POLEmut tumors are associated with excellent prognosis after standard surgical management, this advantage does not clearly translate into superior outcomes during hormonal treatment. Additional biomarkers, including hormone receptor expression, PI3K/AKT/mTOR pathway alterations, and ARID1A, CTNNB1, and ESR1 mutations, may further refine prediction of treatment response within molecular subgroups. Despite its clinical potential, the widespread implementation of molecular classification remains limited by economic and infrastructural barriers, including restricted access to sequencing technologies. Future prospective studies are needed to validate molecular guided fertility sparing strategies and to integrate molecular profiling with additional predictive biomarkers in order to optimize individualized management of young women with EC.

## Introduction

Endometrial cancer (EC) is the most common gynecologic malignancy in developed countries, with an increasing incidence driven by aging populations, obesity, and metabolic syndrome (1, 2). Although it predominantly affects postmenopausal women, a significant proportion of case arises in younger patients, many of whom wish to preserve fertility. Approximately 7% of EC diagnoses occur before the age of 45, making fertility preservation an increasingly relevant clinical challenge (3, 4).

Fertility-sparing treatment (FST), primarily based on progestin therapy, has emerged as a validated alternative to hysterectomy in carefully selected patients with early-stage, low-grade endometrioid carcinoma without myometrial invasiona or atypical endometrial hyperplasia. Despite complete response rates approaching 80%, recurrence remains frequent and difficult to predict, highlighting the limitations of conventional clinicopathologic criteria in guiding patient selection and risk stratification (5).

The introduction of The Cancer Genome Atlas (TCGA) molecular classification has marked a paradigm shift toward a biologically driven understanding of EC (6, 7). Four molecular subtypes were proposed (POLE ultramutated, mismatch repair-deficient (MMRd/MSI-H), p53-abnormal (p53abn), and no specific molecular profile (NSMP)) with a demonstrated prognostic value. POLEmut tumors are characterized by ultramutation and excellent prognosis (8). In contrast, p53abn tumors are driven by TP53 alterations and chromosomal instability, conferring aggressive behavior and poor outcomes; MMRd tumors arise from defects in DNA mismatch repair and are characterized by an intermediate prognosis and NSMP tumors, which represent the majority of EC cases, are biologically heterogeneous (2, 7–10).

Beyond its established prognostic value, molecular classification has the potential to refine clinical decision-making by identifying biologically distinct subgroups that may respond differently to conservative management strategies. However, while the role of the molecular classification in guiding standard surgical and adjuvant treatments is broadly investigated, its application in the fertility-sparing setting remains limited and not standardized. In this context, integrating molecular classification could improve risk stratification, better identify patients who are suitable candidates for conservative management, and potentially inform follow-up strategies and retreatment decisions. The aim of this narrative review is to critically analyze the current evidence on the role of molecular classification in the management of endometrial cancer patients undergoing FST.

## Current strategies and unmet needs

Fertility-sparing treatment is currently reserved for highly selected patients with grade 1, endometrioid EC confined to the endometrium, without myometrial invasion or extrauterine disease. The cornerstone of treatment is progestin-based therapy, administered either systemically (medroxyprogesterone acetate, megestrol acetate) or via levonorgestrel-releasing intrauterine devices, with the goal of inducing tumor regression while preserving reproductive potential (11).

Despite its increasing adoption, FST remains associated with significant uncertainty. Complete response rates are generally high (approximately 75-80%), but not universal, and recurrence is common even after initial remission (2, 5). Moreover, a subset of patients exhibits primary resistance to hormonal therapy, which cannot be reliably predicted using conventional criteria. The limitations of histologic classification are particularly evident in this context and tumor grade and stage do not fully capture biological heterogeneity, and interobserver variability further complicates risk assessment (12). As a result, there is growing interest in identifying biomarkers that can predict response to conservative management and stratify for recurrence risk. Molecular classification may be a promising opportunity to more precisely capturing the genomic landscape of EC and provide a more accurate therapy prediction tool (**Table 1**). A literature search was performed in PubMed/MEDLINE, Scopus, and Web of Science from inception to January 2026 to identify studies evaluating fertility-sparing treatment in endometrial cancer that also provided adequate molecular classification data.

**Table 1:** Summary of studies assessing molecular classification in fertility-sparing management of endometrial cancer.

Study, year	N	Molecular classification method	Molecular group	FST	Oncological outcomes	POLEmut	MMRd / MSI-H	NSMP	p53abn	Median FU
Falcone et al., 2019 (13)	15	ProMisE/NGS panel	POLEmut 1; MMRd 7; NSMP 7; p53abn 0*	Progestin therapy	CR; Recurrence	1/2 (50.0%); 0/1 (0%)	5/6 (83.3%); 1/5 (20.0%)	7/7 (100.0%); 2/7 (28.6%)	NA	106 mo (24-134)
Chung et al., 2021 (12)	57	ProMisE classifier	POLEmut 2; MMRd 9; NSMP 45; p53abn 1	Progestin therapy	CR; Recurrence	1/2 (50.0%); 1/1 (100.0%)	4/9 (44.4%); 1/4 (25.0%)	37/45 (82.2%); 16/37 (43.2%)	1/1 (100.0%); 1/1 (100.0%)	38.4 mo (2.9-163.5)
Puechl et al., 2021 (14)	22	ProMisE classifier	POLEmut 0; MMRd 4; NSMP 17; p53abn 1	LNG-IUS	CR; Progression or definitive treatment;	NA	3/4(75%); 1/4 (25.0%)	13/17(76.5%); 4/17(23.5%)	0/1; 1/1(100%)	28.8 mo (3.3-141.5)
Ran et al., 2022 (15)	12	ProMisE classifier	POLEmut 0; MMRd 3; NSMP 8; p53abn 1	Progestin therapy	CR; Recurrence	NA	2/3 (66.7%); 0/2 (0%)	6/8 (75.0%); 1/6 (16.7%)	1/1 (100.0%); 0/1 (0%)	46 mo (11-138)
Zhang et al., 2024 (16)	59	NGS panel	POLEmut 3; MSI-H/MMRd 4; CNL/NSMP 49; CNH/p53abn 3	Progestin treatment	CR; Recurrence	3/3 (100.0%); NA	1/4 (25.0%); NR	35/49 (71.4%); NR	1/3 (33.3%); NA	16.8 mo (4-104)
Dagher et al., 2023 (17)	20	NGS panel	POLEmut 1; MSI-H/MMRd 3; CN-L/NSMP 16; CN-H/p53abn 0	Progestin therapy	CR; Recurrence	0/1 (0%); NA	0/3 (0%); NA	10/16 (62.5%); 4/10 (40.0%)	NA	48 mo (12-123)
Xu et al., 2023 (18)	90	NGS panel	POLEmut 6; MSI-H/MMRd 5; TP53wt/NSMP 78; TP53abn 1	Progestin therapy	CR; Recurrence	5/6(83.3%); 0/6(0%)	5/5(100%), 3/5(60%)	75/78(96.2%);9/78(12%)	0/1(0%); NA	59.8 (19.1-104.0)
Lv et al., 2024 (19)	93	ProMisE classifier	POLEmut 6; MMRd 15; NSMP/p53wt 67; p53abn 5	LNG-IUS	CR; Recurrence;	6/6 (100.0%); 0/6 (0%)	11/15 (73.3%); 4/11 (36.4%)	57/67 (85.1%); 7/57 (12.3%)	2/5 (40.0%); 2/2 (100.0%)	24 mo
De Vitis et al., 2025 (20)	33	ProMisE classifier	POLEmut 3; MMRd 3; NSMP 25; p53abn 2	Progestin therapy, LNG-IUS, +/- GnRH analog/metformin	CR; Recurrence	2/3 (66.7%); 1/2 (50.0%)	2/3 (66.7%); 2/2 (100.0%)	18/25 (72.0%); 14/18 (77.8%)	1/2 (50.0%); 0/1 (0%)	NA
Wang et al., 2025 (21)	103	ProMisE classifier	POLEmut 3; MMRd 26; NSMP/p53wt 67; p53abn 7	Oral progestins, LNG-IUS, GnRH-analog or combined regimens	CR; Recurrence;	3/3(100%); 2/3(66.7%)	2/3(66.6%); 1/2(50%)	53/67(79.1%) 10/53(19.4%)	4/7(57.1%); 3/4(71.4%)	NA

CR, complete response; EC, endometrial cancer; FU, follow-up; FST, fertility-sparing treatment; GnRH-a, gonadotropin-releasing hormone agonist; LNG-IUS, levonorgestrel intrauterine system; MMRd, mismatch repair deficient; MSI-H, microsatellite instability-high; NA, not available; NGS, next-generation sequencing; NSMP, no specific molecular profile; p53abn, p53 abnormal; POLEmut, POLE mutated; ProMisE, Proactive Molecular Risk Classifier for Endometrial Cancer.

## Molecular classification as a predictor of fertility-sparing outcomes

### POLE mutated group

POLEmut tumors, despite their excellent prognosis in standard management, did not demonstrate superior response rates compared to NSMP tumors in the fertility-sparing setting (5, 13). One of the most interesting findings in the FST setting is the difference between the excellent prognosis of POLEmut tumors with standard treatment and their more modest results with hormonal therapy (18). After standard surgical management, meta-analyses reported a pooled hazard ratio of 0.35 for overall survival and 0.22 for progression-free survival compared to non-POLE tumors (22). This advantage remains even when tumors have features that are usually considered high risk, such as high grade, deep myometrial invasion, or lymphovascular space invasion (22, 23). The biological basis for this excellent prognosis lies in the ultramutated phenotype which causes DNA proofreading loss, resulting in extraordinarily high mutation rates (3, 4). This ultramutation generates a massive neoantigen load, making POLEmut tumors highly visible to the immune system and triggering robust antitumor immunity (3, 4).

Although POLEmut status is a strong favorable prognostic marker after standard surgical management, available fertility-sparing data do not support its use as a reliable predictive marker of progestin sensitivity. While some studies report complete response rates of 66.6-100% for POLEmut tumors, these rates are not significantly superior to NSMP tumors (18, 24). Moreover, POLEmut tumors do not appear to confer the same reduction in recurrence risk observed in surgical series (3). This discrepancy may reflect fundamental differences in treatment mechanisms, as progestin-based therapy primarily acts through hormone receptor–mediated growth inhibition and cellular differentiation, processes that are largely independent of immune surveillance (25, 26). Consequently, the ultramutated phenotype, while associated with increased immunogenicity, does not seem to translate into enhanced progesterone receptor activity or improved hormonal responsiveness (3, 23). Interestingly, some studies suggest POLEmut tumors may achieve complete response more rapidly than other subtypes. Peng et al. reported that POLEmut patients required the shortest median treatment time to achieve complete response compared to MMRd, p53abn, and NSMP (27). The excellent prognosis of POLEmut tumors in standard management should not be extrapolated to predict superior outcomes in fertility-sparing treatment and POLEmut status alone should not be used to expand fertility-sparing criteria beyond current guidelines (20, 28).

### MMRd/MSI-H group

Mismatch repair-deficient tumors consistently demonstrate the poorest response to progestin therapy across multiple studies, with complete response rates of 37.5-48.8% and recurrence rates of 42.8-83.3% (5, 12, 20, 24). These findings suggest that MMRd/MSI-H status may help identify patients with reduced likelihood of durable response to hormonal therapy; however, current evidence does not yet establish it as a definitive predictive biomarker of progestin resistance (29). Multiple studies have demonstrated that MMR deficiency influences hormone receptor expression patterns (12, 30). Okoye et al. found greater variability in estrogen receptor expression in MMRd tumors compared to MMR-proficient tumors, though mean expression levels were similar (30). More importantly, MMRd may affect the functional activity of hormone receptors even when immunohistochemical expression appears preserved. MMRd tumors exhibit distinct epigenetic landscapes that may interfere with progesterone signaling. MLH1 promoter hypermethylation, which accounts for approximately 80% of MMRd cases, is often accompanied by broader epigenetic alterations and this aspect may affect the methylation status of progesterone receptor-binding sites, reducing the transcriptional activity of PR (1, 31, 32). The hypermutated phenotype of MMRd tumors may generate mutations in genes critical for progestin response. The PI3K/AKT/mTOR pathway, which is frequently altered in MMRd tumors and known to interfere with progesterone receptor function, may be further dysregulated by MMR deficiency-associated mutations (33–35). MMRd tumors may present an altered tumor microenvironment that influences hormonal responsiveness, as their increased immune infiltration, although less pronounced than in POLEmut tumors, creates an inflammatory milieu that may paradoxically reduce progesterone sensitivity through mechanisms involving CD163+ macrophages and inflammatory cytokines (36). Zakhour et al. found that none of the patients with abnormal MMR protein expression achieved resolution of hyperplasia or malignancy following progestin treatment, compared to 53% with normal staining (p=0.028) (37). Chung et al. reported that MMRd patients had significantly lower complete response rates at 6 months (11.1% vs 53.3%, p=0.010) and overall response rates (44.4% vs 82.2%, p=0.018) compared to wild-type p53 patients (12). Importantly, MMRd-associated resistance appears to affect not only initial response but also long-term outcomes. Peng et al. demonstrated that MMRd patients had higher relapse rates at 1-year follow-up after achieving complete response (P = 0.035) and higher progression rates at 2, 3, and 4 years (27). While the relationship between MSI-H status and conventional adverse pathological features has not been uniform across studies, MMR deficiency has gained particular clinical importance because it may support sensitivity to immune checkpoint blockade. In current practice, dMMR/MSI-H testing by immunohistochemistry or molecular assays is used both to support Lynch syndrome identification and to select endometrial cancer patients who may benefit from immunotherapy, including anti-PD-1 agents such as dostarlimab (38). In the fertility-sparing setting, this rationale may support the use of PD-1 blockade, alone or combined with progestins, and some ongoing studies are now evaluating this option in selected MMRd/MSI-H patients, particularly when response to standard hormonal therapy is limited (19, 39, 40).

### p-53 abnormal group

p53-abnormal tumors, though rare in the fertility-sparing population, demonstrate consistently poor outcomes with complete response rates of 33.3-75% and recurrence rates of 33-100% (5, 21) The wide range reflects small sample sizes, but the trend toward inferior outcomes is consistent. The biological aggressiveness of p53abn tumors is well-established in standard EC management, where they represent the worst prognostic group (1, 41). In the context of fertility-sparing management, p53abn tumors represent a relatively uncommon but clinically significant subgroup associated with less favorable oncologic outcomes compared with NSMP tumors. In the pooled analysis by Ferrari et al., these tumors demonstrated lower complete response rates, approximately 50%, and relatively high recurrence rates, around 33.3%, suggesting reduced sensitivity to hormonal therapy (5). No significant differences among molecular subtypes were observed in terms of response at 6 months although overall recurrence rates differed across groups with p53abn showing higher recurrence than NSMP. Additionally, p53abn tumors were associated with a significantly higher likelihood of requiring definitive surgical treatment, with increased hysterectomy rates compared with NSMP (p = 0.018) (5). Some evidence indicates that progesterone receptor expression could offer additional insight in this subgroup, as p53abn endometrioid endometrial cancers with retained PR expression have been associated with more favorable progression-free and cancer-specific survival (14, 15, 29). These findings suggest that the subset of p53abn tumors maintaining PR expression might be more amenable to fertility-sparing approaches, although the available data are still limited and require further confirmation (20, 42). Although differences compared with other molecular subtypes are not consistently statistically significant, the overall pattern indicates a less favorable oncologic outcomes, highlighting the importance of careful patient selection and particularly close clinical surveillance when pursuing fertility-sparing approaches. In conclusion, p53abn tumors are well established as an adverse prognostic subgroup in endometrial cancer, and the limited fertility-sparing evidence suggests lower response rates, higher recurrence risk, and a greater likelihood of definitive surgical treatment.

### NSMP group

NSMP tumors represent the ideal candidates for fertility-sparing treatment, consistently demonstrating the highest complete response rates and lowest recurrence rates across multiple studies (14, 15, 18). Studies show that 85-95% of NSMP tumors are ER-positive, significantly higher than other molecular subtypes (43, 44). More importantly, NSMP tumors demonstrate functional hormone receptor signaling, as evidenced by preserved estrogen receptor pathway activity and intact progesterone-mediated gene transcription (43, 44).

These tumors frequently present alterations in the PI3K/AKT/mTOR pathway, particularly PTEN mutations (88%) (8). The pathway determines a paradoxical effect in NSMP tumors, promoting proliferation while also influencing hormonal responsiveness. PTEN loss activates AKT, leading to ligand-independent ERα phosphorylation and sustained ER transcriptional activity, which may preserve hormonal sensitivity despite oncogenic signaling (8, 45). Conversely, hyperactive AKT signaling impairs progesterone receptor function and contributes to progestin resistance (33, 35, 45).Although PTEN loss is common, it does not independently predict survival (46). Different authors reported PTEN loss in 59% of NSMP tumors without prognostic significance, whereas ER-negative tumors showed significantly worse progression-free and overall survival (46, 47).

Vermij et al. showed that ER positivity independently reduced recurrence risk in high-risk NSMP endometrial cancers (HR 0.33, 95% CI 0.15–0.75) (48). Similarly, Jamieson et al. identified low-grade ER-positive NSMP tumors as a very low-risk subgroup with <5% disease-specific mortality at 5 years and outcomes comparable to POLEmut tumors (49).

PR expression is a strong predictor of hormonal therapy response and multiple studies have shown that PR expression >50% is associated with response in advanced or recurrent endometrial cancer in more than half of the analyzed cases (25, 50). In fertility-sparing settings, PR positivity is particularly relevant, as PR-positive tumors respond significantly better to progestin therapy than PR-negative tumors (51).

Progesterone exerts antitumor effects through PR-mediated inhibition of proliferation, induction of differentiation, activation of apoptosis, and suppression of angiogenesis (44, 51, 52). In NSMP tumors, these mechanisms are generally preserved due to sustained ER-driven PR transcription, limited epigenetic silencing compared with MMRd tumors, and intact p53-dependent cell cycle control (12, 16, 53). A recent pooled analysis demonstrated that NSMP tumors represented the majority of fertility-sparing cases (78.8%) and achieved the most favorable oncologic outcomes, with a complete response rate of 78.4% and a recurrence rate of 18.4%, findings consistently reproduced across independent cohorts and supporting the high progestin sensitivity of this subgroup (5). In the same meta-analysis, NSMP tumors showed significantly higher complete response rates and lower hysterectomy rates than MMRd/MSI-H and p53abn tumors (5). Recent evidence indicates that in the NSMP group, estrogen receptor negativity identifies a biologically and clinically distinct subset associated with significantly worse oncologic outcomes compared with ER-positive NSMP tumors, leading to its recognition as a higher-risk category in the updated ESGO risk stratification framework (54). Accordingly, NSMP ER-negative tumors should be approached with particular caution in the fertility-sparing setting and considered separately from ER-positive NSMP disease when evaluating eligibility, counseling, and surveillance strategies (47). Despite their generally favorable outcomes, NSMP status alone should not be interpreted as a predictive biomarker of progestin response, because this subgroup remains biologically heterogeneous. Additional markers, particularly ER/PR expression, and several molecular alterations are likely needed to refine treatment prediction. ARID1A mutations, detected in approximately 56% of NSMP tumors, have been associated with reduced PR expression and potentially decreased sensitivity to progestin therapy (8, 55). Similarly, CTNNB1 mutations may affect hormonal responsiveness through activation of the Wnt/β-catenin pathway, although their specific role in fertility-sparing treatment remains unclear (56, 57). PIK3CA mutations may instead identify patients who could benefit from PI3K-targeted combination strategies, whereas ESR1 mutations could contribute to endocrine resistance (8).

## Economic barriers to implementation and future perspectives

Despite its clinical potential, the widespread implementation of molecular classification remains limited by economic and infrastructural barriers. Comprehensive profiling requires immunohistochemistry, sequencing technologies, specialized laboratory infrastructure, and trained personnel, all of which increase costs and limit accessibility, particularly in low-resource settings (14, 27). Variability in institutional resources and access to next-generation sequencing, especially for POLE testing, has also led to the development of surrogate classification systems based mainly on immunohistochemistry. In addition, the cost-effectiveness of routine molecular testing in low-grade, early-stage tumors eligible for fertility-sparing treatment remains uncertain, supporting the need for selective testing strategies and simplified diagnostic algorithms (1, 2).

Future research should focus on prospectively validating molecular classification in fertility-sparing cohorts and integrating molecular subtypes with additional biomarkers, including hormone receptor expression, pathway activation markers, and epigenetic signatures, to improve patient selection (27, 47).

In this context, validated predictors such as progesterone receptor expression and early Ki-67 suppression may help refine the identification of patients most likely to respond to conservative hormonal treatment (81,88). Several ongoing trials are now directly addressing this transition from a purely histology-based paradigm toward molecularly informed fertility-sparing management (**Table 2**). The PREFACE study (NCT06799624) is prospectively evaluating the prognostic role of molecular classification in women undergoing fertility-sparing treatment for early-stage endometrial cancer, whereas FEMUS (NCT07319429) represents a more interventional step toward subtype-adapted conservative therapy, assigning treatment according to POLEmut, MMRd/MSI-H, and NSMP profiles. In parallel, MMRd-specific strategies are being explored through immune checkpoint blockade, including PD-1 inhibition combined with progestin therapy (NCT06549855) and dostarlimab monotherapy in the SATELLITE trial (NCT06278857). Combination approaches targeting specific molecular vulnerabilities, such as PI3K inhibitors with progestins in NSMP tumors or immunotherapy in MMRd disease, represent promising strategies for improving oncologic outcomes (35, 57, 58). Further studies are also needed to evaluate tailored management approaches for specific subtypes, including less aggressive strategies for POLEmut tumors given their favorable prognosis (3, 23). Emerging technologies, including AI-assisted histopathology, deep learning-based image analysis, and circulating tumor DNA assays, may further support real-time monitoring of treatment response and early detection of recurrence risk (59). However, before these tools can be incorporated into routine fertility-sparing care, future studies must embed prospective biomarker validation, demonstrate reproducibility across laboratories, assess cost-effectiveness, and define standardized algorithms that remain feasible across different healthcare systems (59, 60). Finally, future research should also clarify whether molecular classification can inform not only initial eligibility for fertility-sparing treatment, but also surveillance strategies and post-recurrence management. Although the evidence is still limited, molecular classification may help inform management after recurrence following fertility-sparing treatment. For patients who recur late after a durable complete response and have favorable oncologic and molecular features, a second course of hormonal therapy may be considered after careful reassessment (5). However, even in this apparently lower-risk setting, no validated retreatment algorithm is currently available. The decision to pursue repeat conservative treatment should therefore be individualized, taking into account the timing of recurrence, prior sensitivity to progestins, reproductive plans, and molecular profile. By contrast, early recurrence, poor response to first-line hormonal therapy, p53abn status, or MMRd/MSI-H disease should prompt discussion of definitive surgery or referral to molecularly selected clinical trials, including immunotherapy-based strategies for selected MMRd/MSI-H tumors (17, 20).

**Table 2:** Ongoing trials on fertility-sparing treatment in endometrial cancer integrating molecular classification.

Study	Design	Population	Intervention	Primary endpoints	Molecular stratification	Fertility outcomes reported
Prospective molecular classification cohort (NCT03538704)	Observational, prospective	Patients undergoing fertility-sparing treatment for early-stage endometrial cancer	Any progestin-based regimen	Treatment response	Integrated: POLE, MMRd, p53-abn, NSMP	Fertility outcomes collected
FEMUS (NCT07319429)	Prospective, open-label, multicenter umbrella trial; Phase 2/3	Women 18-45 years with early-stage or selected recurrent EC desiring fertility/uterus preservation	Molecularly tailored therapy: ICI monotherapy for POLEmut; ICI ± progestin for MMRd/MSI-H; MA/MPA or GnRHa + letrozole for selected NSMP	Complete response rate, time to complete response, reproductive and oncologic outcomes	Integrated: POLEmut, MMRd/MSI-H, NSMP; p53-abn excluded; high-risk NSMP features excluded	Fertility and reproductive outcomes explicitly included
PREFACE (NCT06799624)	Prospective observational study	Women 18-45 years with stage IA, grade 1-2 endometrioid EC, no myometrial invasion, desiring fertility preservation	Standard fertility-sparing hormonal treatment according to local practice	Prognostic role of molecular classification for complete response, recurrence and fertility-sparing outcomes	Integrated prognostically: POLEmut, p53-abn, MMRd/MSI-H, NSMP by NGS or ProMisE	Fertility outcomes collected
PD-1 inhibitor + progestin in MMRd early-stage EC (NCT06549855)	Single-arm, open-label interventional study	Women 18-45 years with stage IA, grade 1-2 MMRd endometrial adenocarcinoma	Sintilimab or pembrolizumab combined with progestin therapy	Complete response rate and time to complete response	MMRd-only population	Pregnancy rate, recurrence rate and live birth rate included among secondary outcomes
SATELLITE (NCT06278857)	Phase 2b, open-label, single-arm, multicenter pilot study	Women >=18 years with early-stage MMR-deficient endometrioid EC wishing to preserve uterus or unsuitable for surgery	Dostarlimab monotherapy	Absence of endometrial cancer after protocol treatment; feasibility, safety and efficacy	MMRd-only population	Uterus/fertility preservation included in clinical rationale; fertility outcomes assessed in follow-up

EC, endometrial cancer; FST, fertility-sparing treatment; GnRHa, gonadotropin-releasing hormone agonist; ICI, immune checkpoint inhibitor; MA, megestrol acetate; MMRd, mismatch repair deficient; MPA, medroxyprogesterone acetate; MSI-H, microsatellite instability-high; NGS, next-generation sequencing; NSMP, no specific molecular profile; POLEmut, POLE-mutated; ProMisE, Proactive Molecular Risk Classifier for Endometrial Cancer; p53-abn, p53-abnormal.

## Conclusions

**Figure 1:**
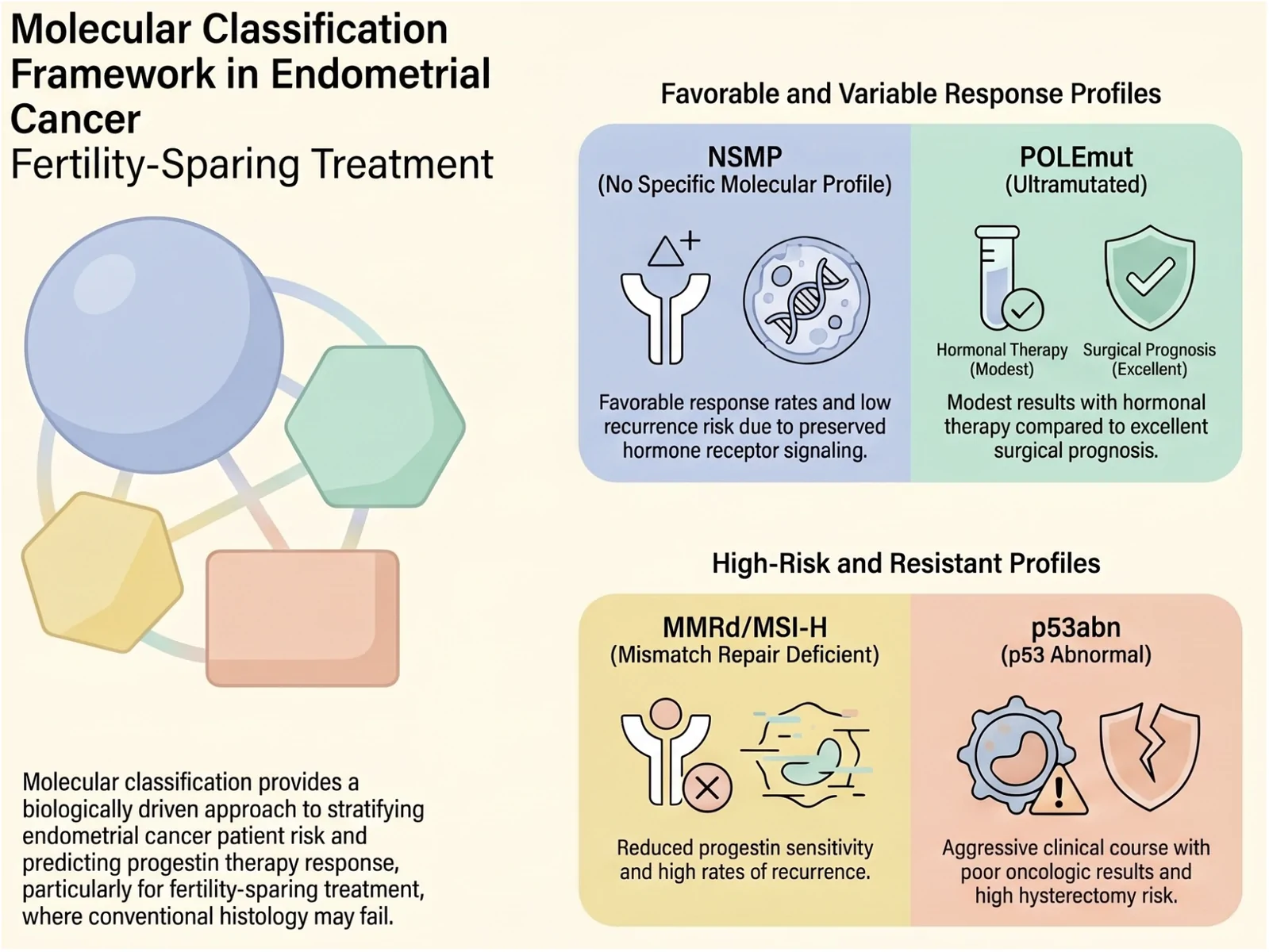
Molecular classification–based risk stratification and predicted response to fertility-sparing treatment in endometrial cancer.

Molecular classification represents a promising tool to improve personalized fertility sparing treatment in endometrial cancer (**Figure 1**). Current evidence suggests that molecular subtypes are associated with distinct patterns of response to hormonal therapy and recurrence risk. NSMP tumors appear to be the most suitable candidates for conservative management, likely due to preserved hormone receptor expression and maintained hormonal signaling. In contrast, MMRd/MSI H and p53abn tumors are associated with poorer oncologic outcomes and may require closer surveillance or alternative therapeutic strategies. Although these findings support the clinical relevance of molecular classification, prospective studies are still needed before its routine integration into fertility sparing management can be fully standardized.
